# Treatment of superficial and deep partial width second degree burn's wound with allogeneic cord blood platelet gel

**DOI:** 10.1111/srt.13471

**Published:** 2023-09-20

**Authors:** Mohammad Ali Nilforoushzadeh, Elham Torkamaniha, Mostafa Dahmardehei, Mohammad Amir Amirkhani, Maryam Heidari‐Kharaji, Parvin Mansouri, Shamim Hortamani, Sona Zare

**Affiliations:** ^1^ Skin and Stem Cell Research Center Tehran University of Medical Sciences Tehran Iran; ^2^ Skin Repair Research Center, Jordan Dermatology and Hair Transplantation Center Tehran Iran; ^3^ Department of Microbial Biotechnology Islamic Azad University Kish Branch Iran; ^4^ Burn Research Center Iran University of Medical Sciences Tehran Iran; ^5^ Stem Cell and Regenerative Medicine Center of Excellence Tehran University of Medical Sciences Tehran Iran; ^6^ Institut National de la Recherche Scientifique (INRS)‐Centre Armand‐Frappier Santé Biotechnologie (CAFSB) Laval Quebec Canada; ^7^ University of British Columbia Faculty of Pharmaceutical Sciences Vancouver Canada; ^8^ Laser Application in Medical Sciences Research Center Shahid Beheshti University of Medical Sciences Tehran Iran; ^9^ Stem Cell and Regenerative Medicine Center Sharif University of Technology Tehran Iran; ^10^ Department of Mechanical Engineering Sharif University of Technology Tehran Iran

**Keywords:** allogenic platelet gel, cord blood, growth factors, regenerative medicine, second‐degree burn wound, wound healing

## Abstract

**Background:**

Burns are caused by a variety of mechanisms, including flames, hot liquids, metallurgy, chemicals, electric current, and ionizing and non‐ionizing radiation. The most significant burn wound management involves complete repair and regeneration as soon as possible while minimizing infection, contraction, and scarring in the damaged tissue area. Some factors such as delivery of nutrients, growth factors, and oxygen are essential to promote and stimulate the wound healing progress in the burns area. When these factors are not provided, the burn wound undergoes a physiological crisis. The use of growth factors is a promising approach to overcoming this limitation. Umbilical cord blood platelet concentrates are a rich natural source of growth factors.

**Methods:**

This clinical trial used growth factors released from the lysis of umbilical cord blood platelet concentrates that have a key role in promoting re‐epithelization and regeneration of damaged tissues by forming a fibrin network. This study evaluated the effectiveness of allogeneic cord blood platelet gel topical dressing in a group of patients diagnosed with superficial and deep partial thickness (second‐degree) burn wounds. Clinical outcomes were compared between the intervention group and a control group of patients with superficial second‐degree burn wounds who received the standard routine treatment including paraffin gauze wound dressing and silver sulfadiazine ointment.

**Results:**

The study's results showed that the increased rate of recovery and tissue granulation completely promoted to wound healing and burn wound closure, decreased the recovery time, and reduced inflammation and scars caused by burn injuries. However, the use of cord blood platelet gel topical dressing is not currently a routine treatment method in patients suffering from burn wounds. However, the study's results showed that allogenic cord blood platelet gel could be used to treat superficial and deep second‐degree burns as a routine treatment. It was also shown that allogenic cord blood platelet gel topical dressing could be a candidate for autograft or after autograft skin transplantation surgery (in donor and recipient sites) instead of skin surgery in some patients.

**Conclusion:**

Allogeneic topical wound dressing provides an effective treatment that offers a faster rate of epithelialization and healing of wounds and also decreases patients’ scar and inflammation level as well as the length of recovery time. This, finally, leads to better burn wound management and the improved quality of burn wound treatment.

## 1 INTRODUCTION

The skin as the body's main protective organ, includes 15% of the body's weight and one and a half to two square meters of the body surface area, and is constantly exposed to a variety of injuries. The skin performs a mixture of functions, including protecting the body against the environment, controlling water entering and leaving the body, providing electrolytes and various substances that protect against various pathogenic microorganisms, preventing ultraviolet rays, and providing mechanical agents that have social and sexual functions.^1^, ^2^ The skin has three distinct layer as follow: the outermost layer is epidermis, the inner layer the is dermis, and the subcutaneous layer is hypodermis. Burns are categorized to four groups; first‐degree, second‐degree, third‐degree, and fourth‐degree. Previously, burns were divided into three categories of first‐, second‐, and third‐degree. However, the new classification includes first‐degree superficial burns (the epidermal layer), which are associated with burning. Superficial partial‐thickness burns involve the entire epidermis layer and the dermis surface (which itself is divided into two surfaces, the superficial partial‐thickness A and the deep partial thickness B). Deep partial thickness or third‐degree burns reach the hypodermis of the subcutaneous layer and, in many cases, are not painful for the patient as they damage the nerves. Fourth‐degree burns reach the bone and tendon tissue and are in fact a kind of necrosis that leads to the loss of the injured limb in most cases.^3^, ^4^ Topical burn care generally includes stopping the burn process, cleaning the injury site according to the type and degree of burn, removing the burn layer according to its depth (debridement), and using topical ointments for healing. The proper burn surface cooling has been shown to have many benefits, including decreasing pain, stopping the tissue necrosis progression that affected by high temperatures, and probably helping heal of the wound.^1^, ^5^, ^6^, ^7^, ^8^

### 1.1 Standard topical care in second degree burn wounds

When a burn injury occurs, it initially damages the skin barrier that is mainly responsible to protect the body from the entry of foreign substances, including pathogens. Infections caused by some fungi and bacteria are the primary causes of morbidity and mortality.^1^, ^2^, ^3^, ^4^, ^9^ A dry, dark scab or falling away of dead skin in the burn wound site provides a suitable environment in terms of temperature, nutrients, and humidity that leads to microbial growth. The first method to prevent infection in the burn wound site is to use topical antimicrobial agents or antibiotic ointments such as bacitracin, mupirocin, and silver sulfadiazine, as well as other aqueous solutions based on silver or aluminum.^9^, ^10^, ^11^, ^12^ The topical treatment of burn wounds includes cleansing debridement damaged tissues and then using routine burn wound dressing. To raise the efficacy of these topical ointments, they need to be applied several times a day that may increase the pain level and interfere with wound healing.^9^, ^10^ Topical wound dressing based on silver due to formation of biofilm in the wound site causes delayed or incomplete re‐epithelialization of skin cells, leading to the formation of scars (high per pigmentation) in the damaged tissue site.^1^, ^10^, ^11^, ^12^ Standard treatment in burn wounds is generally divided into two groups, that is, permanent and temporary topical care. Topical care is discussed in the following.^13^, ^14^, ^15^

### 1.2 Advanced topical wound dressing in second degree burn wounds

#### 1.2.1 Topical foams

Foam dressings are designed to deal with moderate to high levels of wound exudate. They differ in structure and level of absorbency. Simple foam dressings are made with a sponge based on hydro cellular polyurethane. Exudates and liquid substances are taken into the pores of the foam structure through physical absorption.^1^, ^11^, ^16^ Topical foams do not decompose after absorbing wound exudates.^1^, ^17^, ^18^ They range from simple to advanced foams; however, in general, they focus on absorbing wound exudates, remaining non‐adhesive, and keeping moisture in the damaged area.^1^, ^18^

#### 1.2.2 Hydrogels

By delivering moisture to the wound, hydrogel wound dressings create a slightly wet healing environment, which can promote granulation, epithelialization, and autolytic debridement in the damage tissue.^1^, ^19^ One of the major properties of wound dressing based on hydrogel is the use of the high‐water content material that cools the wound and thus leads to pain relief that can last up to 6 h.^20^

#### 1.2.3 Topical dressing based on cell therapy

Because of a lack of donor sites, wide TBSA burns may not have autologous skin available. When autograft is not a good choice, topical wound dressing should be used with some fresh or cryopreserved cells called allogenic grafts (allografts) that are approved for burn healing.^1^, ^21^, ^22^ There are some kinds of allografts derived from human amnion membrane and human skin that are used for different type of wounds such as burns and dystrophic epidermolysis bullosa. Allografts are divided into two groups of cellular and acellular that are screened for some viruses such as HIV, HPV, HCV, HBV, HTLV1, and V2 to prevent the transfer to donors.^23^, ^24^

#### 1.2.4 Topical dressing based on growth factors

There are many types of topical wound dressing based on the use of allogeneic sources growth factors such as recombinant or natural sources. The most prevalent technique to use growth factors for topical wound care is the usage of them in creams, solutions, gels, hydrogels, and cellular and acellular scaffolds.^1^, ^4^, ^23^ Some of growth factors that have key effect in wound treatment are as follow; EGF, FGF, TGF, bFGF, VEGF, and PDGF.^23^, ^25^, ^26^ Growth factors derived from autologous platelet‐rich plasma from patients’ own blood can be sometimes used instead of allogenic and recombinant resources. However, in this clinical trial, we produced and used growth factors derived of umbilical cord blood platelets as a safe allogenic natural resource with the added natural source of thrombin obtained from each cord blood, specifically to prepare fibrin scaffolds. The efficacy of allogeneic topical wound dressing (CBPG) was evaluated in superficial and deep partial thickness (second‐degree) burn wounds.^4^, ^23^, ^25^

## 2 METHODS

### 2.1 Inclusion criteria

Our research team evaluated the efficacy of umbilical cord blood platelet gel in wound healing in two groups of patients. The first group of patients included children with dystrophic epidermolysis bullosa, for whom CBPG dressing was immediately used after pseudosyndactyly surgery. The effectiveness results of this dressing for this group were previously published.^23^ The second group included patients with second‐degree burns, who were discussed in the following. A plastic surgeon and a dermatologist visited the patients, and after a definitive diagnosis of the second‐degree burn, the patients were given umbilical platelet gel derived from cord blood dressing based on the exclusion and inclusion criteria of the study (Table 1). Almost all patients in the both groups were aged between 20 and 40 years, except two patients in the intervention group (Table 1) who were 2 and 4 years old, respectively. These children had burn injuries deeper than the second‐degree burns and their parents did not agree to hospitalization, auto‐skin grafting, or amniotic membrane surgery. Thus, with the consent of the parents and the surgeon, they received CBPG dressing. All patients in the both groups were admitted to the hospital as soon as the burn injuries occurred and did not use any other dressing or medication before this intervention. The control group included four patients with superficial partial thickness (second‐degree) burn wounds. They received routine wound dressing that included silver sulfadiazine and sterile paraffin gauze as global standard (Table 2). The duration of this clinical experiment was between 4 to 16 days for intervention group and 21 to 25 days for the control groups. All patients in both groups included intervention and control did not have any diseases history such as diabetes and blood disorders all of them only had a burn's ulcer. The Consent form from patients and consent to collect cord blood were obtained from patients and donors

**TABLE 1:** Summary of patient characteristics in the intervention group.

No.	Gender	Age (year)	Side of burn's ulcer	Duration of treatment (days)	Number of CBPG per dressing
1	M	40	Left leg	10	2
2	M	39	Right leg	10	2
3	F	36	Right arm	13	2
4	M	21	Right leg	4	1
5	M	35	Right leg	10	2
6	M	40	Left leg	13	2
7	M	22	Right hand	7	2
8*	M	2	4 fingers (whole over and palm) of the right hand	16	2
9*	M	4	Left hand	7	2
10	M	25	Right hand	7	2
11	M	30	Right hand	10	2
12	F	21	Left hand	10	2
13	M	40	Right leg	7	2
14	M	37	Right arm	16	4

**TABLE 2:** Summary of patient characteristics in the control group.

No.	Gender	Age (year)	Side of burn's ulcer	Duration of treatment (days)
1	F	40	Right leg	25
2	F	30	Left arm	25
3	M	22	Right arm	21
4	M	20	Right leg	21

### 2.2 preparation of cord blood platelet gel

The preparation of cord blood platelet gel to use as a topical wound dressing included three steps that are respectively mentioned as follows:

#### 2.2.1 Collection of cord blood

The cord blood units were gathered in hospital after passed the all steps that included the mothers' healthy and informed consent.^23^, ^25^, ^26^, ^27^, ^28^ The consent to collect cord blood were obtained from patients and donors. After each baby was born, the umbilical cord was clamped immediately and wiped with antiseptic. Then, a needle was inserted into the vein in the umbilical cord under sterile condition to withdraw 40 mL blood into a clinical grade blood bag with citrate phosphate dextrose anticoagulant (6.5 mL). A closed system bag (the Noavaran Salamat Arzhang Co., Iran) was used as a standard kit to prepare CBPG by unique designing for 50 cc cord blood.^23^

#### 2.2.2 Preparation of cord blood platelet concentrate (CBPC)

The cord blood units were transferred to the laboratory under sterile condition to prepare CBPC. The cord blood samples evaluated for viral infections like HBV, HPV, HCV, CMV, HIV, and HTLV 1–2.^23^ The units of cord blood were kept at room temperature (RT) up to 48 h. The units were used only after the results of the screening tests were obtained to be negative concerning the viral infections as described previously.^23^, ^26^ The count of platelet for each unit of cord blood should be 120 to 150 × 10^9^/L, also the count of nucleated cell should be >1500 × 10^6^. The reason is that nucleated cells higher than this number for each cord blood unit are appropriate for transplant of hematopoietic stem cell and not for CBPG preparation.^23^, ^26^, ^27^, ^28^ All steps for CBPG preparation were performed in a GMP grade clean room. The bags of cord blood were centrifuged (900 rpm, 15 min) to separate RBC (red blood cells) from the whole plasma. Then, the bags were put in a mechanical extractor by soft pressure to the mechanical handpiece and the whole volume of plasma was moved to new sterile bag that joined to the first blood bag. When counting the number of platelet cells finished, in order to isolated the platelet‐rich plasma of the platelet‐poor plasma all the plasma bags were centrifuged once more (2600 rpm for 15 min). The optimal number of platelets (800 to 1200 × 10^9^/L) and the determined volume of plasma were measured using a special Excel program.^23^, ^25^, ^26^, ^27^, ^28^ Finally, the sterile bags containing CBPC were frozen and stored at −80°C with no cryoprotectant. The CBPC pack can be stored at −80°C for 5 years under aseptic conditions. The platelet‐poor plasma was kept for fungal and bacterial cultures.^23^

#### 2.2.3 Cord blood platelet gel preparation

To examine CBPG on the burn wounds, first, the platelet concentrate pack was thawed with sterile conditions when the platelet concentrate was melted completely. Then, 10% calcium gluconate and natural resources of thrombin were used for gel preparation.^23^ The required thrombin in this clinical trial was collected in sterile tubes without any anticoagulant solution, and the cord blood was collected on the same day. Subsequently, to obtain the blood serum, the clot were centrifuged (4000 rpm, 10–12 min). Finally, the thrombin kept in −80°C.^23^

It should be noted that adding thrombin to CBPC is the key to activate and stimulate the coagulation cascade and form a bio‐scaffold based on a fibrin clot that is defined is a part of the natural process of wound curing in the body. Converting fibrinogen to fibrin by adding thrombin makes fibrin cross linked with a coagulation factor and leads to the formation of a fibrin network.^4^, ^20^, ^23^, ^29^ The amount of thrombin and calcium gluconate depends on the volume of platelet concentrate. In this trial, thrombin and calcium gluconate ratios were one‐fourth and one‐third of the total CBPC volume, respectively. In fact, the amount of thrombin determined the density and consistency of the fibrin network.^23^ The appropriate volume of thrombin was determined according to the volume of platelet concentrate and calcium gluconate. A lower or higher volume of thrombin causes the fibrin network to become loose or dense, respectively, which, in either case, are not suitable for CBPG dressing. The CBPG process was prepared in a GMP grade clean room.^23^, ^25^, ^26^, ^27^, ^28^

#### 2.2.4 CBPG application on the burn wounds

**FIGURE 1:**
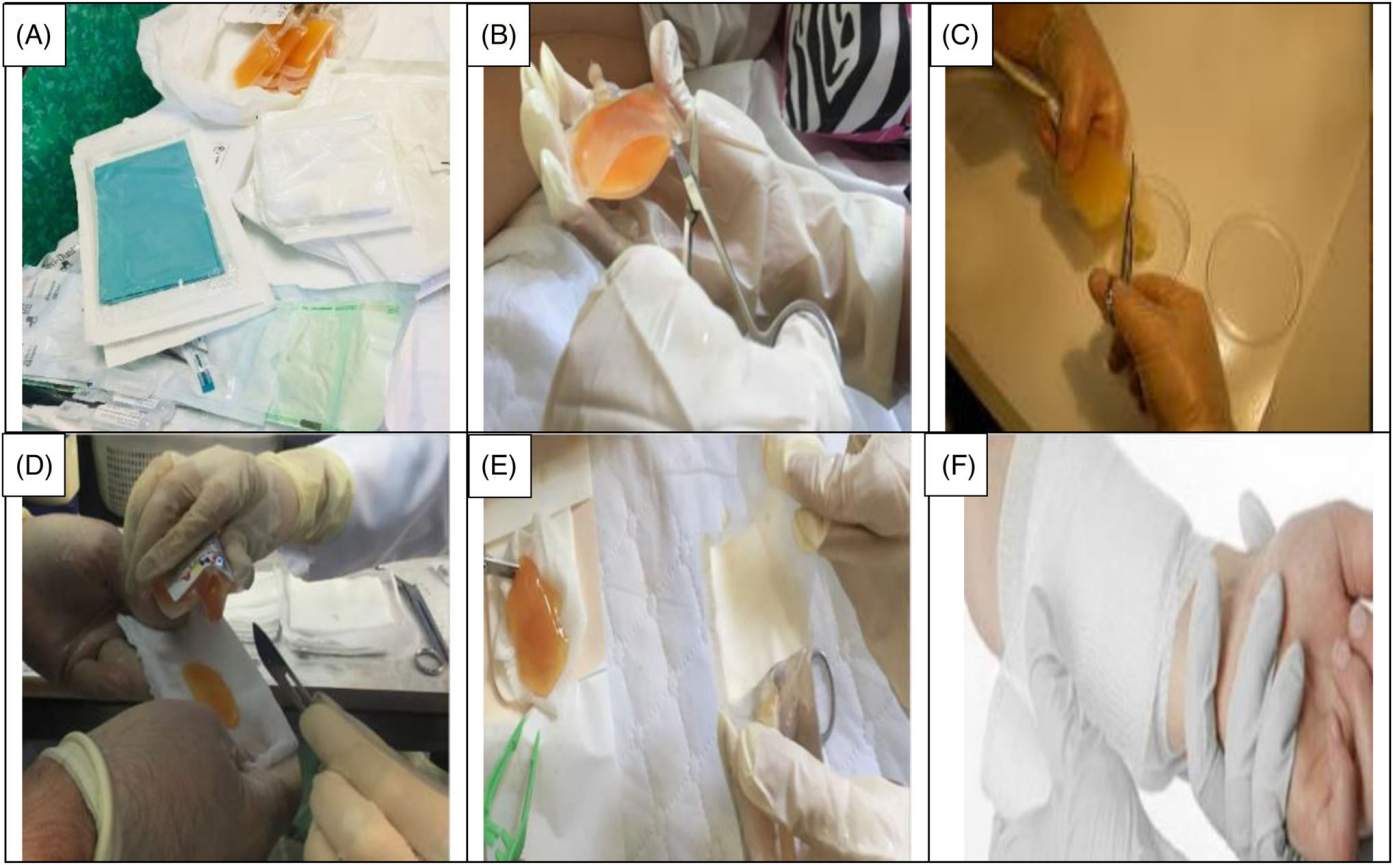
(A) The units of CBPG before application. (B, C) Cut the pack of CBPG with sterile and aseptic conditions. (D, E) Put the CBPG on the paraffin gauze. (F) Apply CBPG topical wound dressing with paraffin gauze on the side of burn ulcer and after bandaged it.

This clinical trial included patients with second‐degree burn wounds. The patients were also candidates of using CBPG for their burn wounds. Small bags of platelet gel (CBPG) were sterilized prior to use.^23^ The first CBPG was used in the wound dressing room once the surface of the burn wounds was cleaned, debrided, and disinfected (day 0). Three or four days after the first intervention (day 3 or 4), wound dressings were opened and changed at the Shahid Motahari Burns Hospital under sterile conditions. At the first, the surface of wound was washed and after the CBPG dressing was opened by normal saline and then aseptic spray was used for sterile the wound area before put the new CBPG dressing. Granulation and re‐epithelialization were assessed on the surface area of the burn wounds in the both group of patients. The second CBPG was applied on the same day. In each session, the wound dressings were examined in the both groups of patients by a plastic surgeon and a dermatologist and the wound healing progress was evaluated. The degree of CBPG use was different among the patients depending on the burn wound size and the tissue regeneration rate. The small CBPG bags were cut from one side using a new surgical blade (bistoury) or a sterile scissor. Then, CBPG was cautiously put on a small pores sterile paraffin gauze dressing to completely preserve the gel and prevent any leaking. The use of paraffin gauze prevents the CBPG absorption by the gauze texture and allows the formation a non‐ adhesive wound dressing on the burn wound site that makes it easier to change the dressing without re‐injury in the intervention site. The topical wound dressing was adjusted and stabilized using bandages (Figure 1A–F). Informed consent was gained from all the patients.

### 2.3 Wound surface area assessment in second‐degree burn wounds

The wound healing and regeneration processes were evaluated by measured the wound surface area (SA) using Image‐J software in the both intervention and control groups following the CBPG use and routine wound dressing including silver sulfadiazine ointment with sterile paraffin gauze application respectively (Tables 3 and 4).^23^

**TABLE 3:** Progression of wound healing in intervention group as a measure of wound surface area (SA) by (Image J) software following per CBPG application for each patient, the rate of wound healing (SA) and the duration time of wound healing (day) in compare to control group that have used routine wound dressing (silver sulfadiazine and sterile paraffine gauze). The *p* value less than 0.05 was considered statistically significant.

Patient	SA Before treating and using the first CBPG (day 0)	SA after using the first CBPG (day 3 or 4)	SA after using the second CBPG (day 7)	SA after using the third CBPG (day 10)	SA after using the fourth CBPG (day 13)	SA after using the fifth CBPG (day 16)	*p* value
1	18.609 cm^2^	10.509 cm^2^	4.002 cm^2^	Wound healing	–	–	<0.05
2	20.315 cm^2^	11.090 cm^2^	5.512 cm^2^	Wound healing	–	–	<0.05
3	28.009 cm^2^	20.868 cm^2^	12.146 cm^2^	4.404 cm^2^	Wound healing	–	<0.05
4	6.224 cm^2^	Wound Healing	–	–	–	–	<0.05
5	16.728 cm^2^	9.309 cm^2^	3.012 cm^2^	wound Healing	–	–	<0.05
6	20.124 cm^2^	11.509 cm^2^	6.946 cm^2^	1.804 cm^2^	Wound Healing	–	<0.05
7	10.189 cm^2^	4.509 cm^2^	Wound Healing	–	–	–	<0.05
8	18.828 cm^2^	13.738 cm^2^	8.909 cm^2^	4.909 cm^2^	2.244 cm^2^	Wound Healing	<0.05
9	11.409 cm^2^	5.009 cm^2^	Wound Healing	–	–	–	<0.05
10	13.216 cm^2^	6.016 cm^2^	Wound Healing	–	–	–	<0.05
11	16.809 cm^2^	10.246 cm^2^	4.869 cm^2^	Wound Healing	–	–	<0.05
12	14.016 cm^2^	7.519 cm^2^	1.039 cm^2^	Wound Healing	–	–	<0.05
13	10.609 cm^2^	4.009 cm^2^	Wound Healing	–	–	–	<0.05
14	40.209 cm^2^	31.004 cm^2^	22.412 cm^2^	12.169 cm^2^	6.586 cm^2^	Wound Healing	<0.05

**TABLE 4:** Progression of wound healing in the control group as a measure of wound surface area (SA) by (Image J) software following per routine wound dressing application for each patient.

Patients	SA before treating and using the first routine dressing (day 0)	SA after using the first routine dressing (day 3 or 4)	SA after using the second dressing routine (day 7)	SA after using the third routine dressing (day 10)	SAafter using the fourth routine dressing (day 13)	SA after using the fifth routine dressing (day 16)	SA after using the sixth routine dressing (day 21)	SA after using the seventh routine dressing (day 25)	*p* value
1	16.828 cm^2^	14.004 cm^2^	11.768 cm2	8.209 cm^2^	5.004 cm^2^	3.109 cm^2^	2.004 cm^2^	Wound Healing	<0.05
2	18.738 cm^2^	15.520 cm^2^	12.246 cm2	9.506 cm^2^	6. 446 cm^2^	4.509 cm^2^	1.044 cm^2^	Wound Healing	<0.05
3	13.216 cm^2^	11.016 cm^2^	7.700 cm2	5. 446 cm^2^	3.210 cm2	1.039 cm^2^	wound Healing	–	<0.05
4	15.216 cm^2^	13.016 cm^2^	9.700 cm2	6. 446 cm^2^	3.210 cm^2^	1.009 cm^2^	Wound Healing	–	<0.05

### 2.4 Granulation tissue assessment in second‐degree burn wounds

After evaluating tissue granulation in all the patients, it was observed that homeostasis was absent after debride and basic tissue regeneration and formation on the surface region of the burn wounds. In the both groups, the surface of the burn wounds was separated to four parts with scoring: (0) no granulation on the wound surface, (1) 1/4 of the wound surface was granulated, (2) 1/2 of the wound surface was granulated, (3) 3/4 of the wound surface were granulated, and (4) complete granulation occurred in the wound site. In each session of dressing changing, the score was determined and compared to that in the previous session (Tables 5 and 6).^23^, ^30^

**TABLE 5:** Score of the granulation tissue (SGT) in the control group. Evaluation of tissue granulation in the intervention group in based on formation and regeneration tissue on the burn ulcer's surface area. The score was determined by compared to the previous changing dressing session in per patient. The rate of tissue granulation in the intervention group was higher than to compared with control group. The STG in each session of the intervention group demonstrated the high efficacy of CBPG in wound regeneration. The *p* value less than 0.05 was considered statistically significant.

Patients	SGT (day 0)	SGT (day 3or 4)	SGT (day 7)	SGT (day 10)	SGT (day 13)	SGT (day 16)
1	0	2	4	–	**–**	**–**
2	0	2	4	–	**–**	**–**
3	0	1	3	4	**–**	**–**
4	0	4	–	–	**–**	**–**
5	0	2	4	–	**–**	**–**
6	0	2	3	4	**–**	**–**
7	0	3	4	–	**–**	**–**
8	0	2	3	4	**–**	**–**
9	0	3	4	–	**–**	**–**
10	0	1	4	–	**–**	**–**
11	0	2	4	–	**–**	**–**
12	0	2	4	–	**–**	**–**
13	0	2	4	–	**–**	**–**
14	0	1	2	4	**–**	**–**

**TABLE 6:** Score of the granulation tissue (SGT) in the control group.

Patients	SGT (day 0)	SGT (day 3 or 4)	SGT (day 7)	SGT (day 10)	SGT (day 13)	SGT (day 16)	SGT (day 21)	SGT (day 25)
1	0	0	1	2	2	3	4	**–**
2	0	1	1	2	3	3	4	**–**
3	0	1	1	2	3	4	–	**–**
4	0	1	2	2	3	4	–	**–**

### 2.5 Vancouver scar scale assessment (VSS) in second‐degree burn wounds

In the current study, scars in the burn wounds were evaluated in the both groups based on VSS that assesses some variables containing height or thickness, vascularity, pigmentation, and pliability. The scores of these variables were given respectively: 1‐vascularize according follow; 0 = Normal, 1 = Pink, 2 = Red, 3 = Purple. 2‐ pigmentation as follow; 0 = Normal 1 = Hypopigmentation, 2 = Hyperpigmentation. 3‐ pliability as follow; 0 = Normal, 1 = Supple, 2 = Yielding, 3 = Firm, 4 = Ropes, 5 = Contracture. 4‐ height or thickness as follow; (0) = Flat, (1) = <2 mm, (2) = 2–5 mm, (3) = >5 mm. All the patients were followed up after 6 months and a total VSS score was given to each of them (Tables 7 and 8).^31^

**TABLE 7:** Evaluation of Vancouver Scar Scale (VSS) in the intervention group. Evaluation of scar score in both group of patients (intervention and control) by vancouver scar scale 6 months after wound healing on the burn ulcers area, the lower total score of VSS in the intervention group compared to control group demonstrated the effectiveness of application CBPG dressing. The VSS is significantly lower than control group (*p* < 0.05).

Patient	Vascularity (0–3)	Pliability (0–5)	Pigmentation (0–2)	Thickness (0–3)	Total score of VSS (0–13)	*p* value
1	1	0	1	0	2	<0.05
2	0	0	1	0	1	<0.05
3	1	1	1	0	3	<0.05
4	0	0	1	0	1	<0.05
5	2	1	0	0	3	<0.05
6	1	1	0	0	2	<0.05
7	0	1	1	0	2	<0.05
8	0	0	0	0	0	<0.05
9	0	0	1	0	1	<0.05
10	1	0	0	1	2	<0.05
11	1	1	0	1	3	<0.05
12	0	1	1	1	3	<0.05
13	0	0	1	1	2	<0.05
14	0	0	1	0	1	<0.05

**TABLE 8:** Evaluation of Vancouver Scar Scale (VSS) in the control group.

Patient	Vascularity (0–2)	Pliability (0–5)	Pigmentation (0–2)	Thickness (0–2)	Total score of VSS (0–13)
1	1	2	2	1	6
2	2	2	1	2	7
3	0	3	1	1	5
4	2	1	1	1	5

### 2.6 Statistical analysis

Statistical analysis was performed using Microsoft Excel 2013 and Prism 8.0 program (Graphpad Software Inc. 2007, USA). The one‐way ANOVA and Student's *t*‐test were used for differences evaluation. A *p*‐value <0.05 was noticed statistically significant.

## 3 RESULTS

**FIGURE 2:**
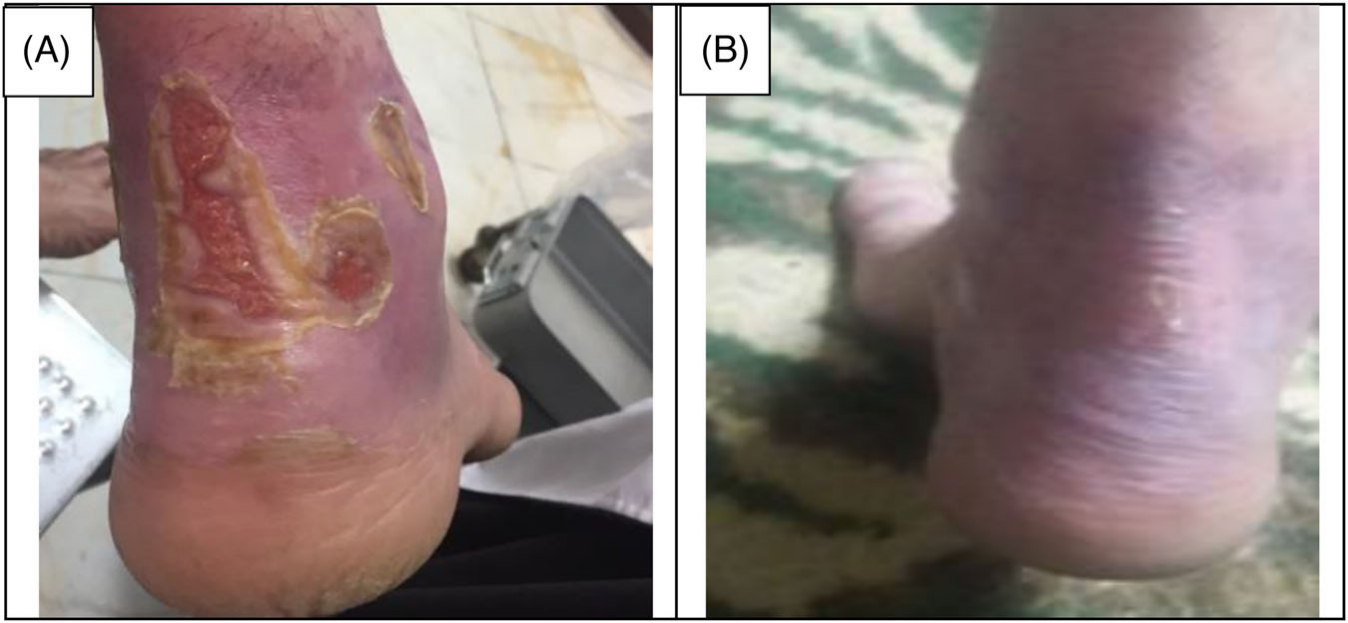
Wound healing process in second burn's ulcer on the Right Leg (patient 4 in Table 1). (A) Before the CBPG application (B) After the first (CBPG) application (4 days from treatment initiation in) and wound healing.

**FIGURE 4:**
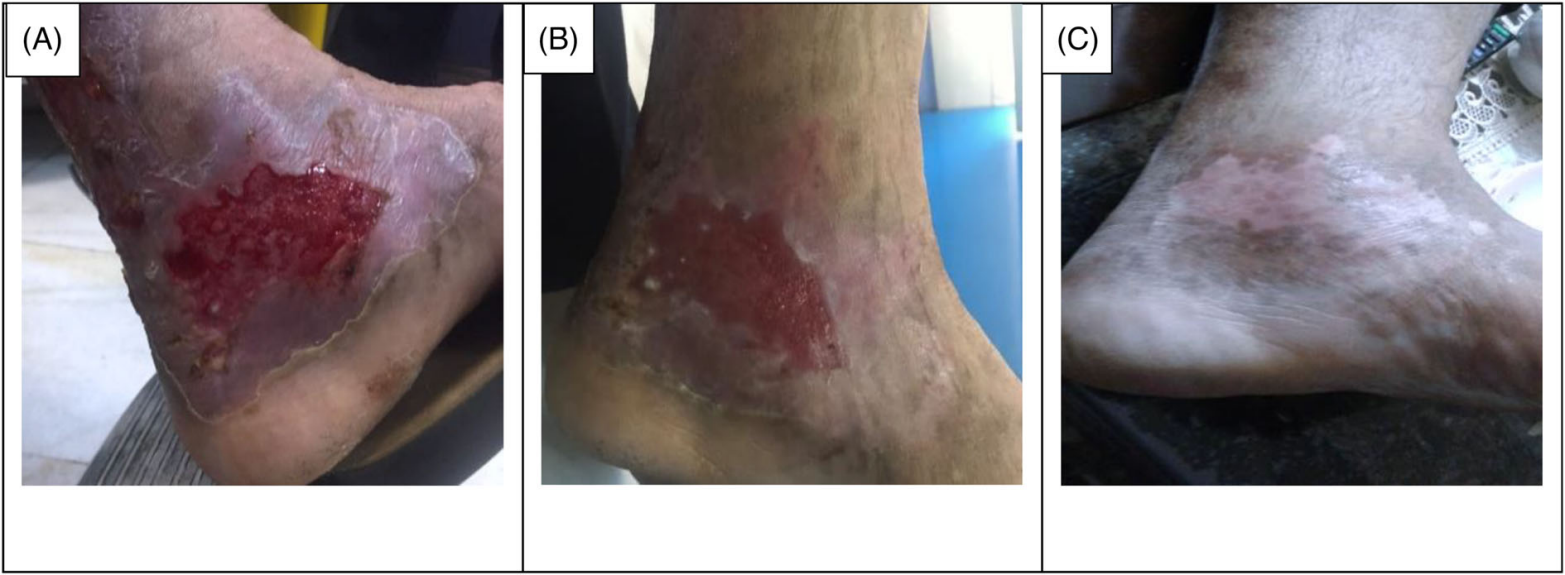
Wound healing process in second burn's ulcer on the Left Leg (patient 13 in Table 1). (A) Before CBPG application (day 0), (B) After the first (CBPG) application (day 4) and (C) After the second (CBPG) application (day 7) and wound healing completely.

**FIGURE 5:**
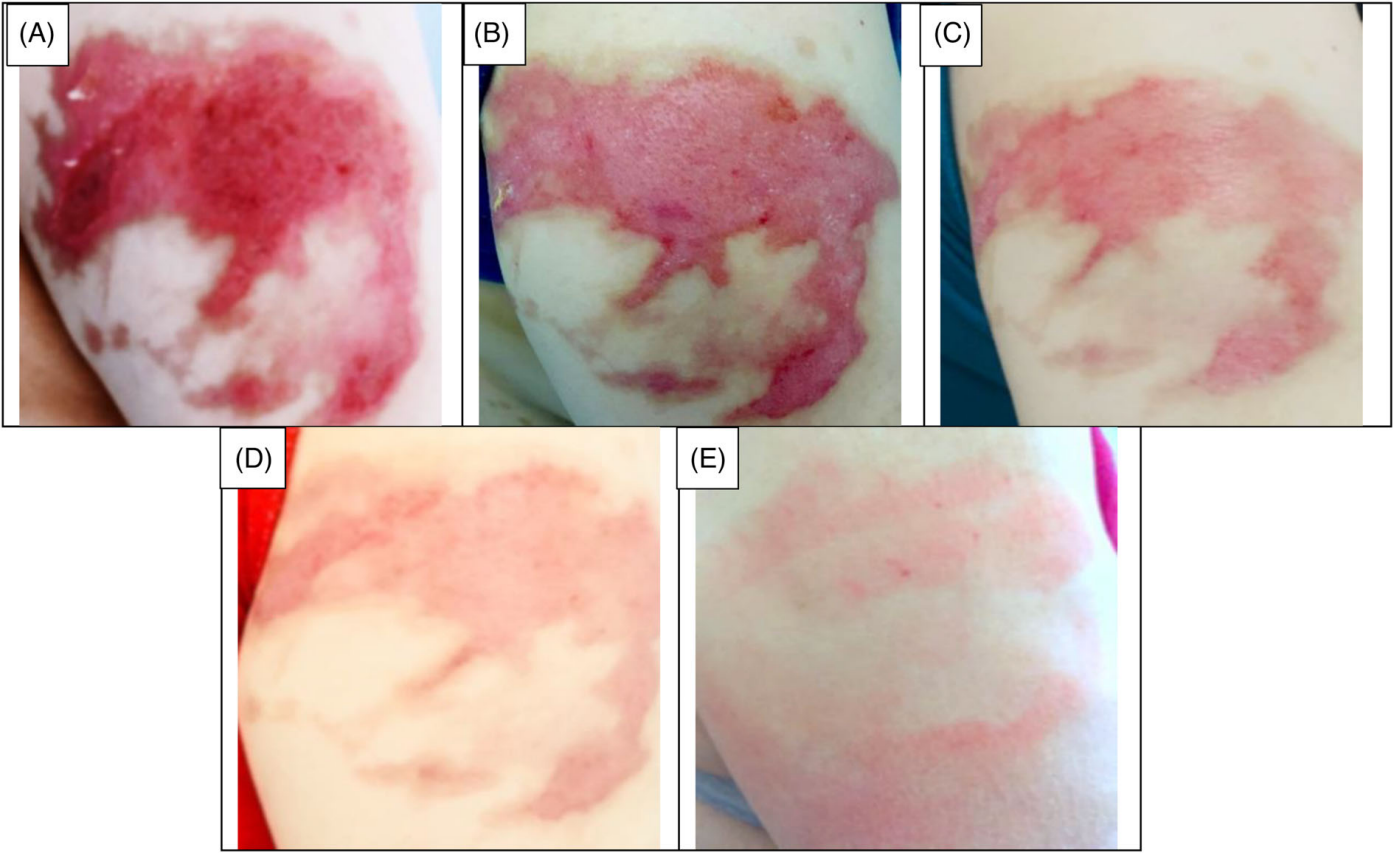
Wound healing in the deep second burn's ulcer on the Right Arm (patient 14 in Table 1). (A) Before CBPG application (day 0), (B) After the first (CBPG) application (day 3), (C) After the second CBPG application (day 7), (D) After the third CBPG application (day 10) and (E) Wound Healing (day 16).

**FIGURE 6:**
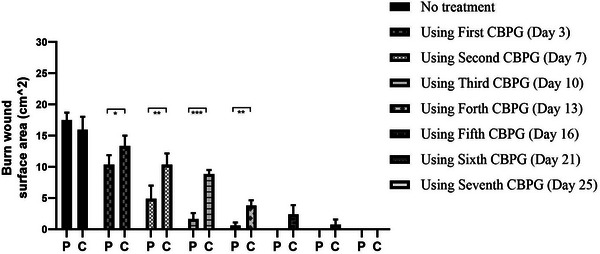
In the intervention group the mean of burn wound surface area was significantly decrease in compare to the control group. P = Patient Group and C = Control Group.

The data collection and the study's follow‐up periods were one year. In this study 18 patients were enrolled as follow; Intervention group include fourteen patients with second‐degree burn wounds (superficial partial thickness) and third‐degree burn wounds (deep partial thickness) and control group include four patients with second‐degree burn wounds (superficial partial thickness). The participants immediately received the intervention after the burn wounds occurred, and they did not receive any treatment previously, as revealed in (Tables 3 and 4). The burn wounds surface area decreased by 6–8 cm^2^ after changing each CBPG dressing regardless of the subjects’ age and gender (Figures 2, 3, 4, 5) Moreover, the control group treated with a routine wound dressing (silver sulfadiazine with paraffine gauze) similar to the intervention group (Table 4), and the burn wounds surface area of this group decreased by around 2–3 cm^2^ after each treatment session. In the intervention group the burn wound surface area was significantly decrease in compare to the control group (Figure 6). In the intervention group regeneration, granulation, and wound healing completely took place in 4–16 days and in the control group 21–25 days, as the depth of the burn wounds was less in the control group than in the intervention group. The recovery and healing process duration varied in the patients based on many factors like wound expansion and depth, the burn wound site location, type of skin, wound management after changing each wound dressing, and stress level. The figures show the entire healing process in four of the patients in the intervention group with different conditions mentioned above (Figures 2, 3, 4, 5) (subjects 4, 8, 13, and 14 in Table 1). Image J software was used to measure the surface area of the burn wounds in the both groups and demonstrate the effectiveness of CBPG in the recovery of injured tissues (Tables 3 and 4). In both groups, the tissue granulation was assessed by dividing the surface of the burn wounds into four parts and observing the granulation progress after changing each topical wound dressing compared with the former session (Tables 5 and 6). The scar level reduced in the intervention group in compared to the control patients, as evaluated using the Vancouver Scar Scale, and they were given a total score of VSS through plus 4 variables assessed such as vascularity, height or thickness, pliability, and pigmentation. Moreover, the highest and lowest VSS scores were 0 and 13, respectively. The total score was given to each patient in the both groups after the follow‐up period of 6 months when the burn wounds healed completely. Accordingly, the total VSS score ranged from 0 to 3 and 5 to 7 in the intervention group and the control group, respectively (Tables 7 and 8). In this study, three parameters including wound surface, granulation tissue, and scar scale were assessed in the two groups. The study's results showed that in the intervention group the CBPG application for the burn wounds increased the rate of recovery, including tissue granulation, regeneration, and complete wound closure, but decreased the scar score, risk of infection, inflammation, and the treatment duration compared with the control group.

FIGURE 3Wound healing process in the deep second burn's ulcer on the Right Hand (patient 8 in Table 1). (A, B) Before CBPG application (day 0), (C, D) After the first (CBPG) application (day 4), (E, F) After the second (CBPG) application (day 7), (G, H) After the third (CBPG) application (day 10), (I, J) After the fourth (CBPG) application (day 13) and (K, L) After the fifth (CBPG) application (day 16) and Wound Healing Completely .

**Figure:**
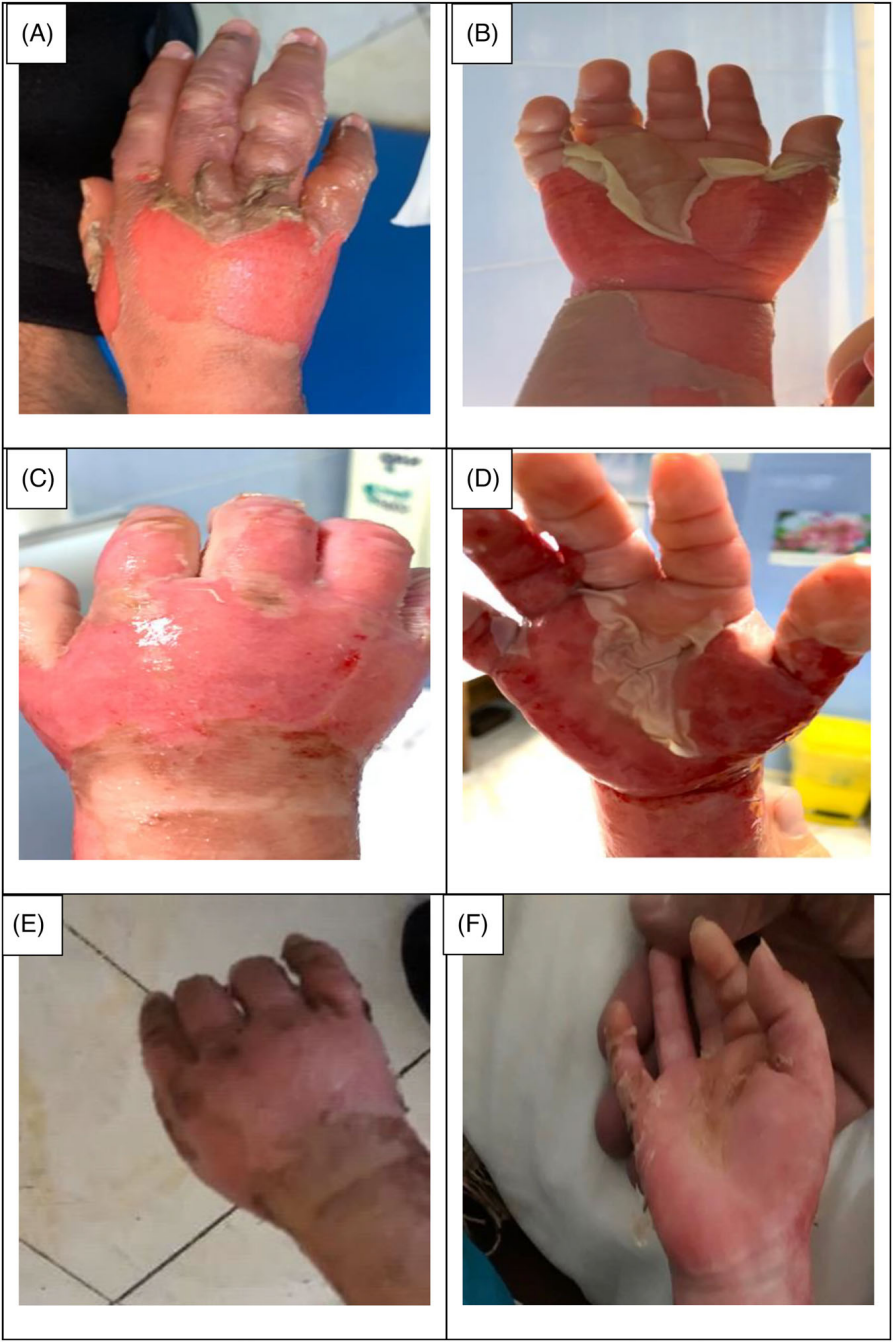

**Figure:**
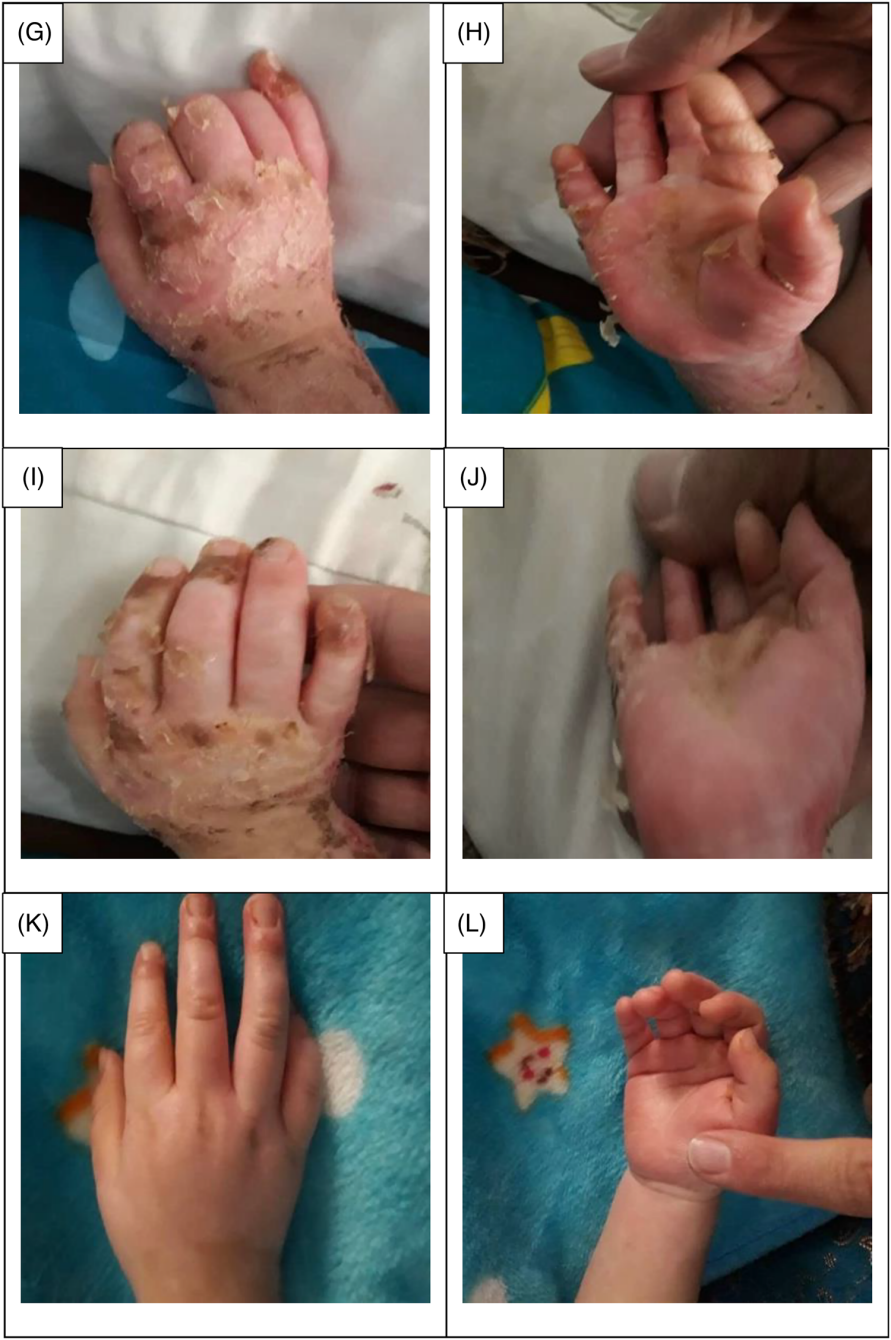

## 4 DISCUSSION

The study's results showed that the usage of allogeneic cord blood platelet gel wound dressing was highly effective in treating superficial and deep partial thickness burn wounds. The results also demonstrated that CBPG could be widely used in the treatment of wounds due to deep second‐degree burns or even in the treatment of patients receiving autologous skin grafts for healing the donor and recipient sites. In some studies, autologous peripheral blood platelet gel was used for wound healing in damaged tissues. However, this has some limitations such as amount of draining blood from patients since some patients may have a blood disorder and patients with burn‐induced stress and pain admitted to the hospital could not tolerate blood sampling and they did not have enough time to wait for the preparation of autologous platelet gel. Moreover, the amount of growth factors released from umbilical cord blood platelets was higher than from allogenic and autologous peripheral blood and also the autologous method was only useable for a small burn wound area. This led to the use of umbilical cord blood platelet gel instead of autologous and allogeneic peripheral blood platelet gel.^23^ CBPG is a natural resource of growth factors compared to topical dressing based on recombinant growth factors. Instead of a factor, CBPG includes a combination of growth factors, each promoting part of the wound healing process. Moreover, CBPG is very cost‐effective compared to the recombinant growth factor wound dressing. In this study, the CBPG efficacy was compared to the allogenic amniotic membrane wound dressing (acellular and cellular) efficacy. The results showed that CBPG topical dressing increased the re‐epithelialization rate and decreased the recovery duration and also it was very effective in (A, B) second‐degree burn wounds in the intervention group (Figures 2, 3, 4, 5). Since acellular allogenic grafts such as amniotic membrane did not have any growth factor to be able to promote and stimulate the wound healing progress. Cord blood platelet gel included higher amount of PDGF as a growth factor that has an effective role in the process of tissue regeneration and wound healing. Further, some growth factors were produce from cord blood platelets, and each factor was effective in each stage of wound healing and damaged tissue regeneration,^23^, ^25^, ^26^, ^27^, ^28^ as briefly discussed below.

PDGF leads to the chemotaxis of neutrophils, macrophages, and fibroblasts, extra cellular matrix remodeling, collagen synthesis, and fibroblast proliferation; TGF‐β stimulates mesenchymal stem cell proliferation and formation, stimulates new extra cellular matrix remodeling, and prohibits macrophages and lymphocyte proliferation. VEGF stimulates angiogenesis that forms new blood vessels and is a factor for promoting vessel permeability; bFGF actively encourages mitosis in fibroblasts, endothelial cells, and mesenchymal stem cells and it generally enhances angiogenesis. EGF raises fibroblast proliferation, migration, and differentiation. In this clinical trial, thrombin derived from cord blood was used instead of batroxobin for gel preparation as a natural, biological resource that is more affordable than batroxobin and other sources of thrombin.^23^ All patients in the intervention and control groups received the first CBPG or routine wound dressing on the same day as the burn occurred and did not receive any other therapeutic dressing previously. Moreover, all the parameters were similarly assessed in the both groups.

## 5 CONCLUSION

CBPG application for superficial and deep partial thickness burn wounds in the intervention group showed that the rate of some parameters increased, which had major effects in the progress of wound healing like granulation, re‐epithelialization, wound surface closure (width and depth), and generally, damaged tissue regeneration. Moreover, the recovery duration, inflammation, infection, and scar level (followed up for 6 months) decreased in the intervention group compared to the control group. All the factors mentioned above facilitated and improved burn wound management. This dressing has the potential to be commercialized and widely used in wound healing because it does not have any immunogenicity reaction at the site of application and also is more effective and cost‐ affordable than cellular and advanced wound dressings.

## CONFLICT OF INTEREST STATEMENT

The authors state no conflicts of interest.

## Data Availability

The data that support the findings of this study are available from the corresponding author upon reasonable request
